# Eubacteria and archaea communities in seven mesophile anaerobic digester plants in Germany

**DOI:** 10.1186/s13068-015-0271-6

**Published:** 2015-06-18

**Authors:** Christian Abendroth, Cristina Vilanova, Thomas Günther, Olaf Luschnig, Manuel Porcar

**Affiliations:** Cavanilles Institute of Biodiversity and Evolutionary Biology, Universitat de València, 46020 Valencia, Spain; Bio H2 Energy GmbH, Im Steinfeld 10, 07751 Jena, Germany; Eurofins Umwelt Ost GmbH, Löbstedter Straße 78, 07749 Jena, Germany; BioEnergie Verbund e.V., Im Steinfeld 10, 07751 Jena, Germany; Fundació General de la Universitat de València, València, Spain

**Keywords:** Biogas, Eubacteria, Archaea, Methanogens, Anaerobic digesters

## Abstract

**Background:**

Only a fraction of the microbial species used for anaerobic digestion in biogas production plants are methanogenic archaea. We have analyzed the taxonomic profiles of eubacteria and archaea, a set of chemical key parameters, and biogas production in samples from nine production plants in seven facilities in Thuringia, Germany, including co-digesters, leach-bed, and sewage sludge treatment plants. Reactors were sampled twice, at a 1-week interval, and three biological replicates were taken in each case.

**Results:**

A complex taxonomic composition was found for both eubacteria and archaea, both of which strongly correlated with digester type. Plant-degrading *Firmicutes* as well as *Bacteroidetes* dominated eubacteria profiles in high biogas-producing co-digesters; whereas *Bacteroidetes* and *Spirochaetes* were the major phyla in leach-bed and sewage sludge digesters. *Methanoculleus* was the dominant archaea genus in co-digesters, whereas *Methanosarcina* and *Methanosaeta* were the most abundant methanogens in leachate from leach-bed and sewage sludge digesters, respectively.

**Conclusions:**

This is one of the most comprehensive characterizations of the microbial communities of biogas-producing facilities. Bacterial profiles exhibited very low variation within replicates, including those of semi-solid samples; and, in general, low variation in time. However, facility type correlated closely with the bacterial profile: each of the three reactor types exhibited a characteristic eubacteria and archaea profile. Digesters operated with solid feedstock, and high biogas production correlated with abundance of plant degraders (*Firmicutes*) and biofilm-forming methanogens (*Methanoculleus* spp.). By contrast, low biogas-producing sewage sludge treatment digesters correlated with high titers of volatile fatty acid-adapted *Methanosaeta* spp.

**Electronic supplementary material:**

The online version of this article (doi:10.1186/s13068-015-0271-6) contains supplementary material, which is available to authorized users.

## Background

Knowledge of the effects of greenhouse gases on the climate dates back to the 1970s, with CO_2_ representing a key greenhouse gas [1]. Today, there is general assent on the urgent need to reduce greenhouse gases in order to mitigate climate change [2, 3]. One of the main strategies to meet this goal requires shifting from fossil to renewable energy sources. In fact, it is expected that by 2020, 20 % of total energy consumption in Europe will be covered by renewable energies [4].

Biomass is a very promising alternative energy source, in particular as a source of biogas. Indeed, almost 70 % of all renewable energies in Europe came from biomass management in 2010 [5], with Germany being a leader in the biomass-based bioeconomy. During recent years, as supported by the EEG (German law for renewable energies) [6], the number of biogas plants and biogas production has increased dramatically in Germany. For example, in 2012, 7200 biogas plants in Germany provided enough energy to power 5.3 million households [7]. Despite this success, the underlying microbial biocenoses of biogas-producing facilities are not yet fully understood, and the whole methanogenesis process is often referred to as a “black box” even in some of the recent literature [7–9]. In the last decades, substantial efforts have been undertaken to shed light on the microbial communities involved in the anaerobic digestion process, as deduced by 16S-rDNA sequencing [10–13], *mcrA* gene-based analysis [14, 15], or metagenomic approaches [16, 17].

Different microbial profiles have been reported for biogas production plants fed with different types of biomass. For example, the microbial diversity in a completely stirred digester fed with fodder beet silage as a monosubstrate is reported to be particularly rich in *Clostridiales*, *Deltaproteobacteria*, *Bacilli*, and *Bacteroidetes* [18]. Other studies describe the effect of biowaste sludge maturation on the microbial profile within a thermophilic digester, which contained mainly *Clostridia* [19]; while the microbial communities in lab-scale reactors fed with casein, starch, and cream are particularly abundant in *Firmicutes* and *Bacteroidetes* [20]. Given these reports, we could say that microbial profiles of anaerobic digesters are, to some extent, specific for each biogas reactor/biomass type. This raises the question whether a common core of microbial key players does exist for anaerobic digesters in general. It is indeed possible to find common microbial actors when higher taxonomic levels are compared. For instance, it is known how methanogenic archaea (genus *Methanosaeta*) dominates environments with low acetate, while increasing amounts of inhibiting substances (like volatile fatty acids or hydrogen sulfide) foster *Methanosarcina* spp. growth [21]. Under thermophilic conditions, *Methanosarcina* spp. proves more frequent than *Methanosaeta* spp. Regarding eubacteria, the phyla *Firmicutes* and *Bacteroidetes* play an important role in anaerobic digestion [13, 22] and within *Firmicutes*, the class *Clostridia* is the most abundant group [18, 23]. Regarding bacteria, and similarly to methanogens stressed above, eubacterial profiles of anaerobic co-digesters and from the anaerobic stage of sewage plants are typically different [13].

**Fig. 1:**
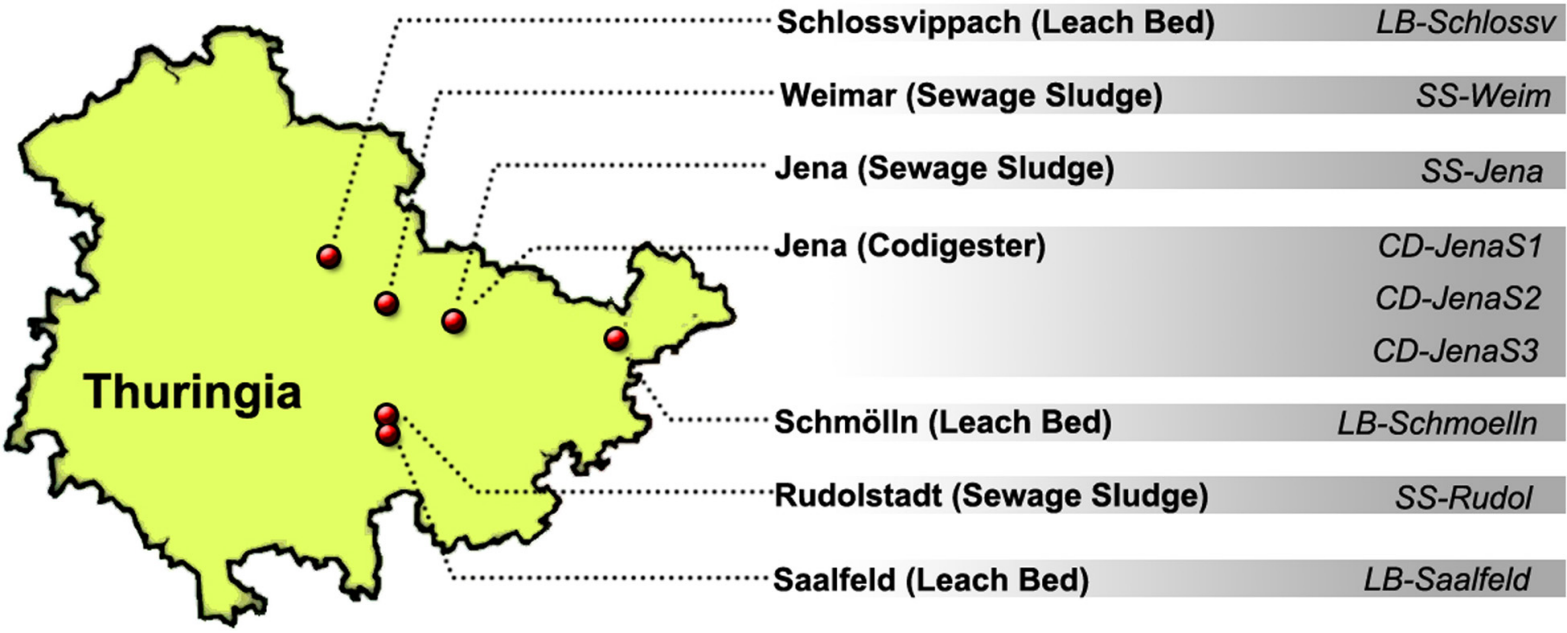
Sampling of anaerobic digesters in Thuringia (Germany). Seven different facilities with a total of nine reactors were sampled in Schlossvippach, Weimar, Jena (two plants, one of them with three reactors), Schmölln, Rudolstadt, and Saalfeld. Sampling was repeated twice at a 1-week interval, and three replicates were processed (54 samples in total). *CD* three-stage plant, *SS* sewage plants, *LB* leach-bed reactors, *S1* plug flow reactor, *S2* continuous stirred tank reactor, *S3* storage tank for digestion remnants

In the present work, we have performed a holistic analysis of seven different digesters at two distinct time points (2 × 9 reactors, sampled within 1 week) from Thuringia, Germany (Fig. 1; Table 1). The digesters corresponded to three different configurations: completely mixed and continuously stirred single-stage tank reactors for sewage sludge digestion (SS); leach-bed digesters operating discontinuously in batches (LB); and a two-stage system consisting of a vertical plug flow reactor followed by an upright continuously stirred tank digester and a final digestate storage tank (hereafter referred to as CD, standing for co-digester). With the exception of the digestate storage tank, which was operated at room temperature (RT), all facilities were operated at mesophilic temperature. The analysis included chemical characterization and biogas measurement of the samples and the determination of the archaea and eubacteria taxonomic profiles by 16S amplicons sequencing on three replicates of each reactor/time. Our results reveal that microbial profiles were strongly dependent on reactor type and moderately dependent on the facility/particular reactor sampled. We also found that profiles were stable in time and exhibited a low degree of variation within the three replicates analyzed. Globally, the 54 subsamples sequenced are the most comprehensive microbial characterization of biogas communities performed to date.

**Table 1:** Overview of sampled digester types and input feeding based on descriptive data

Sample	Digester type	Input materials	Plant configuration
LB-Schmölln	Leach-bed batch digester	Silage, straw, cow manure	Batch process (11 batches)
			Digester volume: 11 × 800 m^3^
			Leachate tank: 1000 m^3^
			Batch process duration: 26–29 days
			Gas production: 0.7 m^3^/m^3^ × day
			OLR: 1.3 kg × VS/m^3^ × day
CD-Jena	Two-stage digester (vertical plug flow reactor/stirred tank)	Silage, farm manure, Livestock farming waste	Two-stage process
			Stage 1 (plug flow): 790 m^3^
			Stage 2 (CSTR): 2000 m^3^
			Stage 3 (final storage tank): 3800 m^3^
			HRT: 87 days
			Gas production: 1.2 m^3^/m^3^ × day
			OLR: 3.0 kg × VS/m^3^ × day
SS-Jena	Completely mixed tank digester	Mono-digestion of municipal sewage sludge	Single stage process (2 digesters)
			Digester volume: 2 × 2000 m^3^
			HRT: 21 days
			Gas production: 0.6 m^3^/m^3^ × day
			OLR: 1.8 kg × VS/m^3^ × day
SS-Weimar	Completely mixed tank digester	Mono-digestion of municipal sewage sludge	Single stage process
			Digester volume: 3200 m^3^
			HRT: 29 days
			Gas production: 0.6 m^3^/m^3^ × day
			OLR: 0.96 kg × VS/m^3^ × day
LB-Schlossvippach	Leach-bed batch digester	Cow manure, straw, *feed residues*	Batch process (8 batches)
			Leachate tank: 1000 m^3^
			Digester volume: 8 × 330 m^3^
			Batch process duration: 32–35 days
			Gas production: 0.5 m^3^/m^3^ × day
			OLR: 2.1 kg × VS/m^3^ × day
SS-Rudolstadt	Completely mixed tank digester	Co-digestion of municipal and industrial sewage sludge with *seasonally available* co-substrates (biodiesel *waste*)	Single-stage process (2 digesters)
			Digester volume: 2 × 2000 m^3^
			HRT: 25 days
			Gas production: 0.3 m^3^/m^3^ × day
			OLR: 0.54 kg × VS/m^3^ × days
LB-Saalfeld	Leach-bed batch digester	Organic fraction of municipal solid waste	Batch process (9 batches)
			Digester volume: 9 × 826 m^3^
			Leachate tank: 1060 m^3^
			Batch process duration: 33 days
			Gas production: 0.7 m^3^/m^3^ × day
			OLR: 0.9 kg × VS/m^3^ × day

Gas production is given in cubic meter of produced gas per cubic meter of sludge per day

*HRT* hydraulic retention time, *CSTR* continuous stirred-tank reactor, *OLR* organic loading rate

## Results and discussion

### Chemical parameters

Eleven parameters were measured for each of the reactor samples: COD (chemical oxygen demand), TOC (total organic carbon), total nitrogen content (N), electrical conductivity, TVFA (total volatile fatty acids), TS (total solids), VS (volatile solids), pH, biogas yield, and concentrations of CH_4_ and CO_2_ (Additional file 1: Table S1). Biogas yields were obtained from lab-scale batch experiments, whereas all the other parameters originated from in situ measurements of digester samples. Batch experiments were performed without adding substrates and obtained biogas yields depended only on the organic fraction within the sludge samples.

**Fig. 2:**
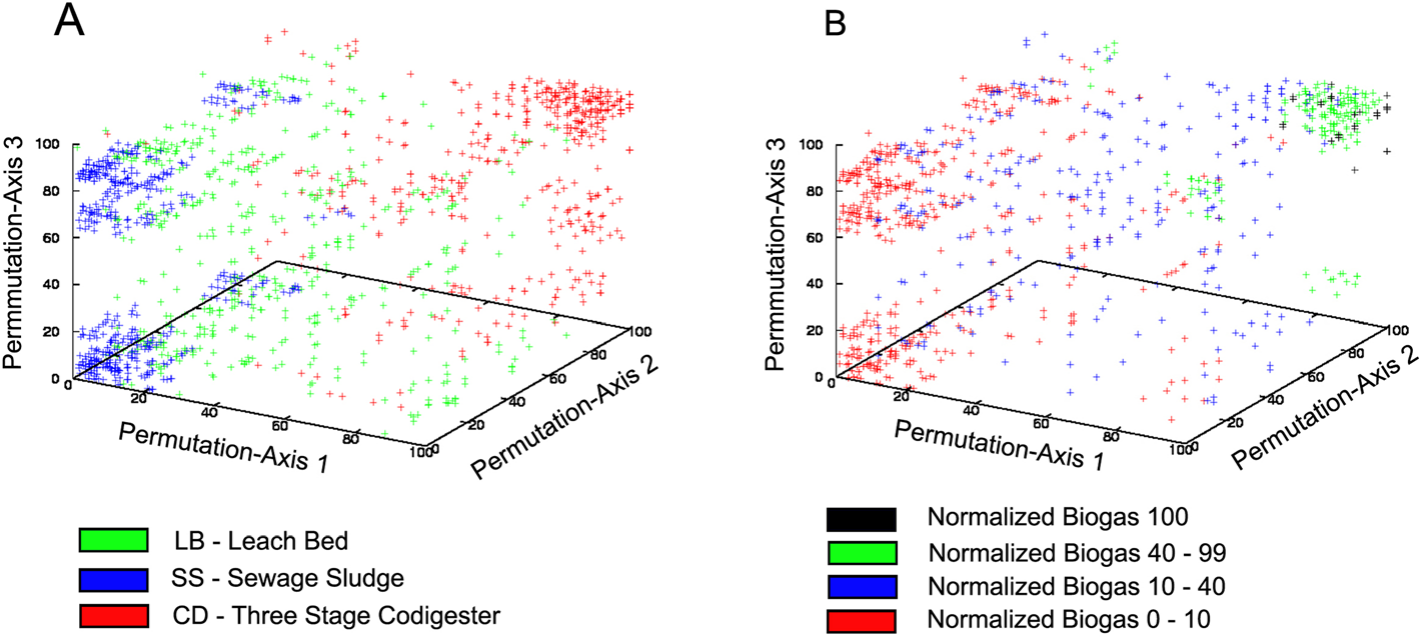
3D plots of chemical parameters. COD, TOC, total nitrogen contents (N), conductivity, TVFA, TS, VS, pH, and produced volume of biogas are plotted in a 3D representation in which the permutation of all determined parameters define axis X, Y, and Z. The underlying biogas facilities are highlighted correspondingly (**a**). Plotting the parameters without the biogas yield and colorizing the *dots* according to their biogas production rate gives the second plot (**b**)

After normalizing the data, successive combinations of three parameters (permutation) were plotted in a Gnuplot multiplot (Fig. 2). The resulting data matrix included biogas production but not methane and CO_2_ concentration, in order to avoid redundancies. This resulted in three clearly defined clouds, each corresponding to one of the different digester facility types (Fig. 2a). SS and CD values were plotted in two opposed vertices of the plot, with LB located in an intermediate position. The yield of biogas produced is shown in Fig. 2b and the highest yields are plotted as a relatively small cloud (black dots) overlapping with the extremes of the CD cloud. As a general conclusion, parameter values were higher (corresponding in general with high nutrient contents) when biogas production was highest. In a second statistical approach, this observation was verified by a principal component analysis (Additional file 2: Figure S1), where samples coming from the same type or reactor clustered together and notably differed from those from other reactor types.

### Taxonomic composition of eubacteria

**Fig. 3:**
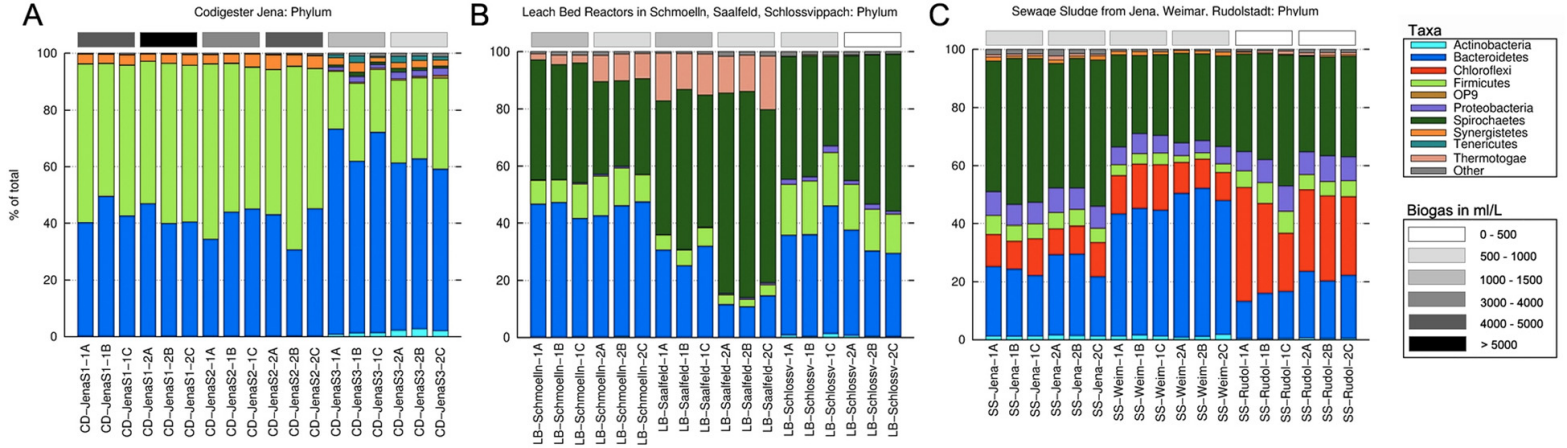
Bacterial profiles of the anaerobic digester plants analyzed. Taxonomic (phylum) composition of eubacteria populations in the reactors as deduced by 16S amplicons isolated and sequenced as described in “Material and methods” section. **a** Three-stage co-digester (CD) plant in Jena, (**b**) leach-bed reactors, and (**c**) sewage plants. The *grey scale* (*top right*) corresponds to biogas yield ranges as shown at the *right*

Eubacteria from all samples were identified by high-throughput sequencing as described in “Material and methods” section, and phylum-level results are shown in Fig. 3. There was little variation between replicates, clearly indicating that differences in taxonomic composition accounted for the differences found between reactors and time. Similarly, different sampling times resulted in very small variations in the taxonomic profile, being the taxonomic composition of each sample very constant after 1 week. Only in one case (LB reactor in Saalfeld) that a substantial shift was detected in the amount of *Bacteroidetes* and *Spirochaetes* after 1 week.

The taxonomic composition of the samples correlated closely with reactor type. Indeed, three different profiles were observed, each corresponding to a particular facility type. CD samples were dominated by the phylum *Firmicutes*, with nearly 46–60 % of classified sequences assigned to *Firmicutes* in the first two stages and less than 20–32 % in the third stage (remnant storage); followed by *Bacteroidetes*, which proved mainly in the third stage, when it accounted for up to 73 % of the total identified taxa. The three CD digesters contained low amounts of *Synergistetes*, and the remnant storage contained moderated amounts of *Actinobacteria*, *Proteobacteria*, *Spirochaetes*, and *Tenericutes* (Fig. 3a).

The second facility type (LB) displayed a totally different microbial composition (Fig. 3b) with comparatively fewer *Firmicutes* reads (between 3 and 19 % of total sequences). The microbial LB communities were dominated by *Spirochaetes* (30 and 72 % of the total reads), along with *Bacteroidete*s (11 and 47 %). The third phylum, *Thermothogae*, reached low to moderate frequencies in LB facilities in Schmölln and Saalfeld (between 2 and 19 %), and it was absent in the six replicates of Schlossvippach. Minor counts of *Actinobacteria* and *Proteobacteria* were also detected. The third profile was associated with the sewage sludge digesters (Fig. 3c). Although the SS facilities showed certain similarities compared to the LB facilities, the overall microbial composition differed from both CD and LB reactors. In common with the LB samples, SS reactors contained high amounts of *Bacteroidetes* and *Spirochaetes* (*Bacteroidetes* between 13 and 51 %, *Spirochaetes* between 27 and 50 %). However, unlike the CD and LB facilities, SS reactors were particularly rich in *Chloroflexi* (9 and 39 %) and *Proteobacteria* (4–9 %). Besides the aforementioned taxa, small amounts of *Actinobacteria*, *Synergistetes*, and *Thermotogae* were also observed.

Minor variations or sub-profiles of the three main biomass-associated profiles were detected. For example, two of the three Jena CD reactors were very similar, while the third one displayed higher eubacteria diversity. This might be due to the fact that the last stage (remnants) was kept at RT instead of mesophile temperatures. Although LB and SS samples corresponded to two main profiles, one location of each type (LB-Schlossvippach and SS-Rudolstadt) exhibited a characteristic presence/absence of one particular taxon: the former typically lacked *Thermotogae*, which was well represented in the other two LB plants; while SS Rudolstadt was particularly rich in *Chloroflexi* (Fig. 3b, c). The absence of *Thermotogae* in the LB reactor from Schlossvippach may be due to the fact that the solid phase is mainly heated up by the leachate (without extra heating in the solids storage—“garage”), which can lead to irregularities in temperature. In the Schlossvippach sample, it took more than 1 week to heat up a newly filled garage (Christoph Bürger and Kevin Lindner personal communication).

In general, taxonomic eubacteria profiles strongly correlated with the biomass type. The differences observed between CD and SS reactors are in accordance with previous studies [13] describing an overall difference between sewage sludge and co-fermentation regarding the microbial profile. The high amount of *Bacteroidetes* and *Firmicutes* in CD reactors is also consistent with previous reports [13, 18, 22, 24]. One reason for the abundance of *Firmicutes* could be the high content in TS derived from plant material (Additional file 1: Table S1), which probably fosters biofilm formation. *Firmicutes* have been described as main degraders of cellulolytic material [24] and are abundant in biofilms of water supply systems [25, 26]. LB and SS reactors, both containing liquid substrates, had high titers of the very mobile and efficient swimmer *Spirochaete*, described as able to swim in high viscous gel-like liquids, such as those found in LB reactors [27]. It has to be highlighted that the observed microbial profiles for the LB samples were only those from leachate, and that the solid fraction of LB systems might be rich in *Firmicutes* due to the high percentage of solids. The abundance of *Chloroflexi* in SS reactors has previously been reported. In fact, different *Chloroflexi* species have been found in more than 60 sewage reactors in different European countries based on FISH experiments [28] and also in other facilities around the world [29]. The prevalence of *Proteobacteria* and *Bacteroidetes* is in accordance with the work by Wang et al*.* [30] on the microbial profile of domestic sewage outfalls.

The different taxonomic profiles we found correlated to biogas yield. For instance, the phylum *Chloroflexi* was detected in sewage plants, where very low biogas yields were measured. Also, *Proteobacteria* were only found in the plants with low biogas yields (digestate storage of the three-stage plant, Schlossvippach, and all sewage samples), while *Firmicutes* were particularly abundant in reactors with high biogas yields (CD samples). However, differences in biogas yield might also be a consequence of the concentration of TS, which is especially high in CD reactors.

In summary, our results are strongly consistent with previous reports demonstrating patchiness of the digesters in terms of the distribution of bacterial populations [31]. This strongly suggests ecological parameters (i.e*.* liquid/solid substrate or biomass type) are the key factors shaping microbial communities; but also reveal an important, albeit secondary, role of the facility/reactor on this mainly biomass-associated distribution of the taxonomic profiles.

### Taxonomic composition of archaea

**Fig. 4:**
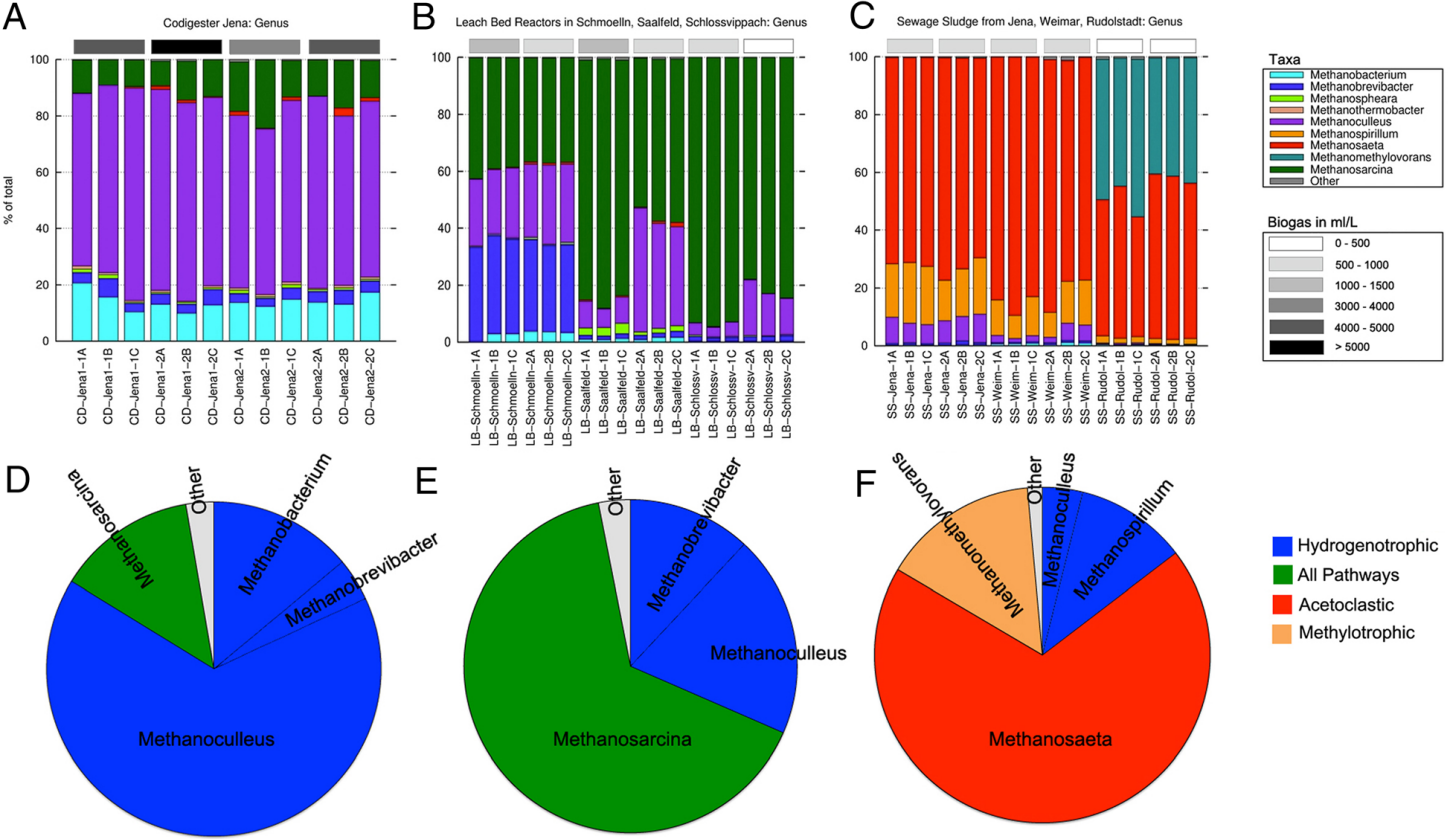
Taxonomic (genus) composition of archaea in the anaerobic digester plants. Taxonomic composition based on 16S archaea-specific amplicon sequences is shown. **a** The three-stage plant (CD) in Jena, (**b**) leach-bed reactors, and (**c**) sewage plants. The *grey scale* (*top right*) corresponds to biogas production values as in Fig. 2. Samples corresponding to the storage tank of the digestion remnants reactor (CD-Jena S3) are not shown as they failed to produce any amplicon with the selected oligonucleotides. Methanogenesis pathways are shown in (**d**) three stage plant, (**e**) leach-bed reactors, and (**f**) sewage plants

The taxonomic composition of the sampled reactors in terms of archaea contents is shown in Fig. 4. The data correspond to all but one reactor (three replicates and two time points), corresponding to the third stage of the Jena CD reactor, from which no archaeal DNA could be amplified. CD reactors were dominated by archaea belonging to the genus *Methanoculleus* (Fig. 4a), accounting for 59–76 % of all the sequences. A significant amount of *Methanosarcina* (9–24 %), *Methanobacterium* (10–21 %), and *Methanobrevibacter* (3–7 %) was detected, as well as infrequent genera such as *Methanosphaera*, *Methanothermobacter*, and *Methanosaeta*. In contrast, LB digesters were characterized by substantially smaller amounts of *Methanoculleus* (3–44 %); and by the abundance of *Methanosarcina* (37–95 %). One of the three LB-digesters showed a very high amount of *Methanobrevibacter* (31–35 %), whereas the other two reactors had very low amounts (1–2 %). Minor genera were *Methanobacterium*, *Methanosphaera*, and *Methanosaeta*. In the SS samples, *Methanosaeta* proved the most prevalent genus with a total number of reads between 42 and 88 % (Fig. 4c). While *Methanosaeta* was detected in high amounts in all the SS reactors, the frequency of other genera differed among SS digesters. The biogas plant in Rudolstadt was very rich in *Methanomethylovorans* (40–55 %), while the other two SS reactors showed a relatively high amount of *Methanoculleus* (1–10 %) and *Methanospirillum* (8–21 %).

As in the eubacteria profiles, three main taxonomic combinations were found to correlate with the three reactor types. The CD samples showed a strikingly similar profile independently on the replicate, reactor, or time sampled. LB and SS reactors did exhibit sub-profiles with no variation within replicates and dependent on the sampling time (Schlossvippach and Saalfeld) or on the location sampled (Rudolstadt). The two LB facilities from Schlossvippach and Saalfeld showed an increased amount of *Methanoculleus* after 1 week, while the amount of *Methanosarcina* decreased during this period. It is likely that genus *Methanoculleus* is more abundant in the solid fraction of these LB systems due to the high percentage of solids. Rudolstadt samples had the typical *Methanosaeta* abundance of SS reactors but were characterized by an exceptionally high frequency of *Methanomethylovorans*. The presence of *Methanosarcina* and *Methanoculleus* correlated to high yields of biogas, while low biogas yields correlated with higher amounts of *Methanosaeta*.

Since methane production is solely due to the archaeal community and the different methanogenesis pathways are well known and genus-linked, we studied the expected methanogenesis pathways in each facility type according to the average taxonomic distribution (Fig. 4d–f). Interestingly, each facility type displayed a different combination of methanogenesis pathways. The CD reactors were very rich in archaea using the hydrogenotrophic pathway (Fig. 4d); LB reactors were dominated by *Methanosarcina* and thus with the ability to use all known pathways for methane production (Fig. 4e); and SS reactors were characterized by containing high rates of archaea using the acetoclastic pathway for methane production (Fig. 4f).

The archaea composition we describe here for the different reactor types is generally in accordance with that reported in previous studies. The prevalence of *Methanoculleus* in CD reactors was also found in other works with classical anaerobic digesters [22, 32, 33]. Although other studies describe a prevalence of *Methanosarcina* in this reactor type [34], our data is in concordance with other works linking *Methanosarcina* to LB reactors [35, 36]. The differences in TS levels between CD and LB reactors might be the key factor explaining their differences in microbial composition. The TS content of LB reactors was much lower (Additional file 1: Table S1), so the surface available for the growth of biofilm-forming species, such as *Methanoculleus* [37], was limited compared to CD reactors. Indeed, previous reports have found a prevalence of *Methanoculleus* in the solid fraction of LB reactors [36, 38]. Additionally, a lower number of TS may hamper the formation of spatial syntrophic relationships between acetate-oxidizing bacteria and hydrogenotrophic methanogens such as *Methanoculleus*. This might lead to an increase in growth of acetoclastic methanogens such as *Methanosarcina,* able to directly metabolize acetate (Fig. 4d–f). These findings are in concordance with previous reports on the link between high content of TS and a high frequency of hydrogen-using methanogens compared to acetoclastic methanogens [39–42].

The finding that *Methanosaeta* is the dominating genus in all SS digesters is consistent with other screenings [21, 43, 44]. However, the abundance of *Methanomethylovorans* in the SS digester in Rudolstadt might be connected to the presence of particularly high amounts of oil and alcohols such as methanol, since this particular digester was supplemented with remnants from biodiesel production, and the prevalence of this organism has been reported in sewage sludge reactors supplemented with molasses alcohol wastewater [45].

The genus *Methanospirillum* was more abundant in the SS reactors in Jena and Weimar but not in Rudolstadt. This genus proved, along with *Methanolinea*, particularly abundant in a previous SS characterization [46], suggesting that *Methanospirillum* and *Methanosaeta* are two competing genera within the anaerobic digestion process of SS sludge.

## Conclusions

The present work describes a holistic characterization of, to the best of our knowledge, the widest screening of biogas production facilities performed to date. We studied nine reactors, three replicates, and two time points (1-week interval) yielding 54 subsamples, the taxonomic diversity of which was determined for both archaea and eubacteria contents. Despite the heterogenous nature of some of the samples (especially those from CD reactors), our data reveal a very small effect of inter-replicate variation. All our results suggest a strong link between reactor type and taxonomic profile (for both archaea and bacteria), as well as an additional, significant effect of the location/particular reactor on the microbial community. Additionally, the three reactor types yielded separate blocks when chemical parameters were plotted in 3D and a principal component analysis was performed. Taken together, our results confirm the tight link between digester type, chemical parameters, and microbial biocenoses and also support the existence of a very stable microbial core adapted to each reactor type. Furthermore, our study provides a strong dataset for future diagnostic strategies aiming to predict biogas production of mesophile reactors on the basis of their microbial composition.

## Materials and methods

### Sampling

Seven anaerobic reactors accounting for nine different reactors from Thuringia, Germany, were sampled twice at a 1-week interval. These biogas plants included co-digesters, leach bed, and sewage sludge treatment plants (Fig. 1). Triplet samples from the first sampling time point were labelled as 1A, 1B, and 1C; whereas triplet samples from the second time point were labelled as 2A, 2B, and 2C.

An overview of the sampled digester types and input feedings is shown in Table 1. Additional file 1: Table S1 and Additional file 3: Table S2 show specific environmental chemical parameters regarding biogas production, biogas composition, and VFA spectrum. All sampled digester types were operated at mesophilic temperature (except the sampled storage chamber for digestion remnants, which was left at RT). For the chemical analysis, a total volume of 5 L was collected in buckets via a sampling port at each plant. The sampling procedure was similar for all plants and stages (SS plants, LB systems, and all the stages of the one-phase CD). In the case of the LB facilities, only leachate from the leach tank could be collected. Small amounts of sample were then transferred into Falcon tubes, which were directly frozen on dry ice to prevent further microbial growth or DNA degradation, and immediately sent on dry ice from Thuringia to Valencia (Spain) for DNA isolation and sequencing. The remaining sludge was transferred to the laboratory of Bio H2 Energy GmbH in Jena. From this sludge, 1.5 L was used for gas production analysis directly upon sampling. The remaining 3.5 L of sludge was aliquoted into smaller plastic boxes and stored at −20 °C for further analysis at Eurofins and Bio H2 companies.

### Determination of biogas production

For each anaerobic sludge sample, 1.5 L was incubated in batch-experiments for 1 week at 37 °C. Incubation bottles (0.5 L) were filled with 0.5 L of sample (three bottles per sample without additional feeding), connected to a liquid displacement device (eudiometer, custom-built model calibrated by the German Eichamt), and the whole setup was flushed with nitrogen to ensure an anaerobic atmosphere. Biogas yield was measured as produced volume of biogas per volume of sludge sample [mL/L]. The concentration of CO_2_ and CH_4_ in the produced biogas was determined with the “Binder COMBIMASS GA-m” gas-measurement device (Binder, Germany).

### Measurement of chemical parameters

Totals solids (TS), volatile solids (VS), chemical oxygen demand (COD), electrical conductivity, and total organic carbon (TOC) were determined according to German standard measurement methods [47]. Total nitrogen was determined as previously described (VDLUFA-Methodenbuch II, 3.5.2.7). The VFA spectrum was determined with a gas chromatograph (Shimadzu, Japan). The flame ionization detector was equipped with a DB.1701 column (Machery-Nagel/Germany).

### DNA extraction from reactor samples

Three DNA samples were prepared from each sludge sample. In order to reduce the amount of inhibiting substances (especially humic acids), biomass was sedimented by centrifugation (5–10 min at 20,000 g for SS and LB samples, and 15 min at 20,000 g for CD samples) and washed several times with sterile PBS buffer until a clear supernatant was observed. DNA was isolated with the “PowerSoil DNA isolation KIT” (Mo Bio Laboratories, USA) following the manufacturer’s instructions. Long centrifugations were performed (5–10 min at 20,000 g for SS and LB samples, and 15 min at 20,000 g for CD samples) to ensure an almost complete removal of particles and cell fragments after the mechanical bead treatment.

Finally, DNA quality was checked on a 0.8 % (*w*/*v*) agarose gel and quantified with Nanodrop-1000 Spectrophotometer (Thermo Scientific, Wilmington, DE, USA).

### PCR amplification

In order to survey bacterial diversity, a 500-bp fragment of the V1-V3 hypervariable region of the 16S ribosomal RNA gene was PCR-amplified from all the samples with universal primers 28F (5′-GAG TTT GAT CNT GGC TCA G-3′) and 519R (5′-GTN TTA CNG CGG CKG CTG-3′). In the case of archaea, primers Arch349F (5′-GYG CAS CAG KCG MGA AW-3′) and Ar9r (5′-CCC GCC AAT TCC TTT AAG TTTC-3′) were used to amplify a 578-bp fragment of the 16S region [48]. A short (10–12 nucleotides) barcode sequence was included at the 5′ end of the oligonucleotides used as forward primers in order to assign sequences to samples after high-throughput sequencing. All the amplifications were performed under the following thermal cycling conditions: initial denaturing at 95 °C for 5 min, followed by 35 cycles of denaturing at 95 °C for 30 s, annealing at 54 °C (for both, bacteria and archaea) for 30 s, and extension at 72 °C for 1 min, finalized by a 10-min elongation at 72 °C. The resulting amplicons were checked on a 0.8 % (*w*/*v*) agarose gel and purified by precipitation with 3 M potassium acetate (pH = 5) and isopropanol. Pure amplicons were quantified with the Qubit® 2.0 Fluorometer (Invitrogen, Carlsbad, CA, USA), and two equimolar pools of bacteria and archaea amplicons, respectively, were prepared from all the samples.

### Ion torrent sequencing

Two sequencing libraries were constructed with 100 ng of the eubacteria and archaea amplicon pool, respectively, by the amplicon fusion method (Ion Plus Fragment Library Kit, MAN0006846, Life Technologies). Each library was quantified with the Agilent2100 Bioanalyzer (Agilent Technologies Inc, Palo Alto, CA, USA) prior to clonal amplification. Emulsion PCRs were carried out applying the Ion PGM Template OT2 400 kit as described in the user guide (MAN0007218, Revision 3.0 Life Technologies) provided by the manufacturer. Finally, the libraries were sequenced in an Ion 318 Chip v2 on a Personal Genome Machine (PGM) (IonTorrentTM, Life Technologies) at Life Sequencing S.L. (Life Sequencing, Valencia, Spain), using the Ion PGM Sequencing 400 kit following the manufacturer’s protocol (publication number MAN0007242, revision 2.0, Life Technologies). Sequence statistics are shown in Additional file 4: Table S3.

### Sequence analysis and taxonomic classification

Raw sequences obtained from the sequencing center were processed with the MOTHUR software [49]. Short (<100 bp) and low-quality (<q15) reads were removed in a first step. The degenerated forward primer sequence was searched among the resulting sequences, and reads were discarded if either the primer (three mismatches allowed) or the barcode sequence was missing. Sequences were then split into groups based on barcode matches, and both primer and barcode sequences were trimmed. Finally, each resulting sequence was aligned to the ribosomal 16S reference Greengenes database and taxonomy was assigned based on nucleotide similarity with the k-mer algorithm. Assignments based on a similarity percentage lower than 70 % were not considered for further analysis.

### Statistics

A principal component analysis (PCA) was performed using the Statgraphics software. Data from COD, TOC, total nitrogen contents (N), conductivity, TVFA, TS, VS, pH, and biogas corresponding to all samples were normalized, and two components explaining almost 90 % of the total variance were used for plotting. Row-stacked histograms, representing taxonomic profiles (Figs. 3 and 4), were prepared using Gnuplot and modified with Photoshop to insert grey bars representing intervals of biogas production. Pie charts (Fig. 4) were plotted in Excel.

In order to plot all environmental chemical parameters in one diagram (Fig. 2), the *splot* and *multiplot* commands of Gnuplot were combined to plot the permutation of all normalized parameters (normalized to values between 0 and 100). Each combination with three chosen variables was plotted and overlaid with the other combinations using the Gnuplot multiplot command. Since nine parameters were measured (COD, TOC, total nitrogen contents, conductivity, TVFA, TS, VS, pH, and volume of biogas), 84 resulting combinations were overlaid in the plot (Fig. 2a).

## Additional files

Additional file 1: Table S1. Chemical environmental parameters of analyzed sludge samples and volume and composition of produced biogas (*w*/*o* VFA, error ± 10 %). Additional file 2: Figure S1. Principal component analysis (PCA) performed on the chemical environmental parameters measured for all samples. Data were normalized, and two components explaining nearly 90 % of the total variance were used for plotting. Additional file 3: Table S2. Content of VFA in the sampled reactors (error ± 10 %). Additional file 4: Table S3. Sequencing statistics.

## Abbreviations

AD: Anaerobic digestion
ADS: Anaerobic digester sludge
CD: Co-digester
COD: Chemical oxygen demand
LB: Leach bed
TOC: Total organic carbon
TS: Total solids
TVFA: Total volatile fatty acids
VFA: Volatile fatty acids
VS: Volatile solids
