# Supplementary material for: Eubacteria and archaea communities in seven mesophile anaerobic digester plants in Germany
Source: Biotechnol Biofuels. 2015 Jun 18;8:87. doi: 10.1186/s13068-015-0271-6 (PMC4474353; doi:10.1186/s13068-015-0271-6)
Supplement: Additional file 1: Table S1. — Chemical environmental parameters of analyzed sludge samples and volume and composition of produced biogas (w/o VFA, error ± 10 %). [file 13068_2015_271_MOESM1_ESM.docx]

**Table S1.** Chemical environmental parameters of analyzed sludge samples and volume and composition of produced biogas (w/o VFA, error ± 10%).

|  | **COD**  (KgO_2_/tVS) | **σ**  (mS/cm) | **TOC**  (kg/tVS) | **Total-N**  (kg/tVS) | **pH** |
| --- | --- | --- | --- | --- | --- |
| **LB-Schmoelln-1** | 35,00 | 41,20 | 15,80 | 3,80 | 8,26 |
| **LB-Schmoelln-2** | 34,00 | 20,50 | 29,80 | 3,10 | 8,36 |
| **CD-JenaS1-1** | 135,00 | 35,00 | 72,60 | 7,00 | 8,43 |
| **CD-JenaS1-2** | 144,00 | 36,90 | 77,20 | 8,30 | 8,00 |
| **CD-JenaS2-1** | 129,00 | 42,20 | 63,90 | 7,60 | 8,50 |
| **CD-JenaS2-2** | 139,00 | 40,60 | 67,30 | 7,70 | 8,39 |
| **CD-JenaS3-1** | 103,00 | 32,80 | 64,70 | 7,60 | 8,36 |
| **CD-JenaS3-2** | 88,00 | 37,00 | 34,30 | 4,70 | 8,35 |
| **SS-Jena-1** | 7,00 | 8,90 | 1,70 | 0,90 | 7,50 |
| **SS-Jena-2** | 10,00 | 8,50 | 2,30 | 0,90 | 7,50 |
| **SS-Weim-1** | 8,00 | 9,20 | 2,20 | 1,00 | 7,60 |
| **SS-Weim-2** | 7,00 | 9,10 | 2,20 | 1,00 | 7,40 |
| **LB-Schlossv-1** | 28,00 | 39,70 | 13,30 | 2,90 | 8,00 |
| **LB-Schlossv-2** | 24,00 | 38,60 | 11,00 | 2,50 | 8,30 |
| **SS-Rudol-1** | 5,00 | 5,20 | 1,30 | 0,50 | 7,30 |
| **SS-Rudol-2** | 5,00 | 5,30 | 1,50 | 0,50 | 7,30 |
| **LB-Saalfeld-1** | 25,00 | 43,70 | 11,10 | 3,50 | 8,15 |
| **LB-Saalfeld-2** | 12,00 | 27,60 | 5,60 | 1,70 | 7,88 |
|  |  |  |  |  |  |
|  | **TS**  (%) | **VS**  (% of TS) | **Biogas**  (in mL/L) | **CH_4_**  (%) | **CO2**  (%) |
| **LB-Schmoelln-1** | 3,51 | 56,21 | 1.302,80 | 63,8 | 36,2 |
| **LB-Schmoelln-2** | 3,38 | 55,08 | 751,80 | 55,6 | 44,4 |
| **CD-JenaS1-1** | 17,01 | 73,50 | 4.783,10 | 50,8 | 49,3 |
| **CD-JenaS1-2** | 17,78 | 73,99 | 8.269,70 | 57,3 | 42,7 |
| **CD-JenaS2-1** | 18,56 | 74,42 | 3.376,90 | 48,6 | 51,4 |
| **CD-JenaS2-2** | 15,50 | 71,19 | 4.207,10 | 55,3 | 44,7 |
| **CD-JenaS3-1** | 17,04 | 77,40 | 1.073,10 | 21,0 | 79,1 |
| **CD-JenaS3-2** | 10,62 | 68,64 | 955,40 | 46,4 | 53,6 |
| **SS-Jena-1** | 3,44 | 55,19 | 533,80 | 45,9 | 54,1 |
| **SS-Jena-2** | 3,59 | 57,41 | 548,80 | 62,1 | 37,9 |
| **SS-Weim-1** | 4,11 | 56,01 | 654,40 | 65,8 | 34,2 |
| **SS-Weim-2** | 3,70 | 57,57 | 658,80 | 69,2 | 30,8 |
| **LB-Schlossv-1** | 3,15 | 51,45 | 534,40 | 73,6 | 26,4 |
| **LB-Schlossv-2** | 3,01 | 49,41 | 166,00 | 65,1 | 34,9 |
| **SS-Rudol-1** | 1,20 | 52,70 | 420,90 | 66,5 | 33,5 |
| **SS-Rudol-2** | 1,94 | 50,16 | 226,70 | 69,5 | 33,4 |
| **LB-Saalfeld-1** | 2,45 | 53,41 | 1.060,90 | 69,5 | 30,5 |
| **LB-Saalfeld-2** | 1,52 | 51,34 | 998,60 | 68,9 | 31,1 |
