# Supplementary figures and images for: Eubacteria and archaea communities in seven mesophile anaerobic digester plants in Germany
Source: Biotechnol Biofuels. 2015 Jun 18;8:87. doi: 10.1186/s13068-015-0271-6 (PMC4474353; doi:10.1186/s13068-015-0271-6)

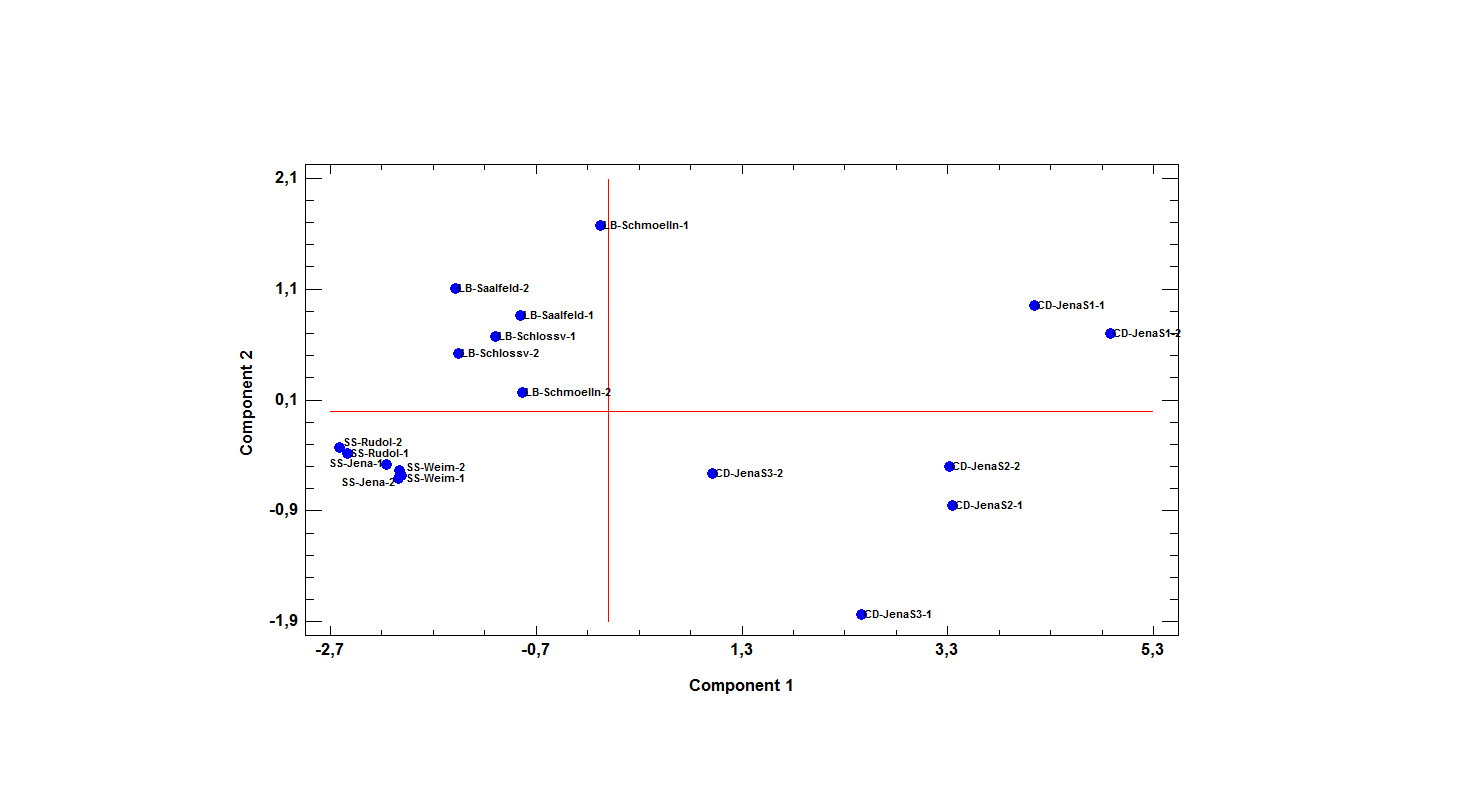

Supplement: Additional file 2: Figure S1. — Principal component analysis (PCA) performed on the chemical environmental parameters measured for all samples. Data were normalized, and two components explaining nearly 90 % of the total variance were used for plotting. [file 13068_2015_271_MOESM2_ESM.png]
