# Supplementary material for: Eubacteria and archaea communities in seven mesophile anaerobic digester plants in Germany
Source: Biotechnol Biofuels. 2015 Jun 18;8:87. doi: 10.1186/s13068-015-0271-6 (PMC4474353; doi:10.1186/s13068-015-0271-6)
Supplement: Additional file 3: Table S2. — Content of VFA in the sampled reactors (error ± 10 %). [file 13068_2015_271_MOESM3_ESM.docx]

**Table S2.** Content of VFA in the sampled reactors (error ± 10%).

|  | **TVFA**  (mg/L) | **Acetic**  **acid** (mg/L) | **Propionic**  **acid** (mg/L) | **Isobutyric acid** (mg/L) | **Isovaleric**  **acid** (mg/L) |
| --- | --- | --- | --- | --- | --- |
| **LB-Schmoelln-1** | 2.010,00 | 1.300,00 | 710,00 | 0,00 | 0,00 |
| **LB-Schmoelln-2** | 747,00 | 690,00 | 57,00 | 0,00 | 0,00 |
| **CD-JenaS1-1** | 3.500,00 | 930,00 | 1.900,00 | 310,00 | 360,00 |
| **CD-JenaS1-2** | 2.670,00 | 870,00 | 1.500,00 | 0,00 | 300,00 |
| **CD-JenaS2-1** | 780,00 | 560,00 | 57,00 | 53,00 | 110,00 |
| **CD-JenaS2-2** | 880,00 | 710,00 | 60,00 | 0,00 | 110,00 |
| **CD-JenaS3-1** | 79,00 | 0,00 | 0,00 | 0,00 | 0,00 |
| **CD-JenaS3-2** | 490,00 | 490,00 | 0,00 | 0,00 | 0,00 |
| **SS-Jena-1** | 0,00 | 0,00 | 0,00 | 0,00 | 0,00 |
| **SS-Jena-2** | 0,00 | 0,00 | 0,00 | 0,00 | 0,00 |
| **SS-Weim-1** | 0,00 | 0,00 | 0,00 | 0,00 | 0,00 |
| **SS-Weim-2** | 0,00 | 0,00 | 0,00 | 0,00 | 0,00 |
| **LB-Schlossv-1** | 300,00 | 0,00 | 0,00 | 0,00 | 0,00 |
| **LB-Schlossv-2** | 0,00 | 0,00 | 0,00 | 0,00 | 0,00 |
| **SS-Rudol-1** | 0,00 | 0,00 | 0,00 | 0,00 | 0,00 |
| **SS-Rudol-2** | 0,00 | 0,00 | 0,00 | 0,00 | 0,00 |
| **LB-Saalfeld-1** | 385,00 | 320,00 | 65,00 | 0,00 | 0,00 |
| **LB-Saalfeld-2** | 1.170,00 | 990,00 | 180,00 | 0,00 | 0,00 |
