# Supplementary material for: Eubacteria and archaea communities in seven mesophile anaerobic digester plants in Germany
Source: Biotechnol Biofuels. 2015 Jun 18;8:87. doi: 10.1186/s13068-015-0271-6 (PMC4474353; doi:10.1186/s13068-015-0271-6)
Supplement: Additional file 4: Table S3. — Sequencing statistics. [file 13068_2015_271_MOESM4_ESM.docx]

**Table S3.** Sequencing statistics.

|  | **Number of Sequences Analyzed for**  **Archaea (bp)** | **Average Length**  **(bp)** | **Number of Sequences Analyzed for**  **Bacteria (bp)** | **Average-Length**  **(bp)** |
| --- | --- | --- | --- | --- |
| **CD-Jena-S1-1A** | 9.511 | 552 | 25.526 | 527 |
| **CD-Jena-S1-1B** | 124.529 |  | 19.867 | 531 |
| **CD-Jena-S1-1C** | 8.088 | 501 | 28.422 | 532 |
| **CD-Jena-S1-2A** | 7.926 | 510 | 18.945 | 528 |
| **CD-Jena-S1-2B** | 10.375 | 501 | 32.839 | 531 |
| **CD-Jena-S1-2C** | 6.394 | 507 | 11.600 | 531 |
| **CD-Jena-S2-1A** | 15.546 | 541 | 6.243 | 539 |
| **CD-Jena-S2-1B** | 17.978 | 541 | 8.116 | 535 |
| **CD-Jena-S2-1C** | 8.907 | 508 | 8.173 | 532 |
| **CD-Jena-S2-2A** | 4.805 | 546 | 5.163 | 529 |
| **CD-Jena-S2-2B** | 2.045 | 482 | 12.872 | 529 |
| **CD-Jena-S2-2C** | 3.441 | 429 | 22.541 | 526 |
| **CD-Jena-S3-1A** | - | - | 36.139 | 526 |
| **CD-Jena-S3-1B** | - | - | 27.249 | 528 |
| **CD-Jena-S3-1C** | - | - | 8.324 | 518 |
| **CD-Jena-S3-2A** | - | - | 22.862 | 514 |
| **CD-Jena-S3-1B** | - | - | 8.381 | 514 |
| **CD-Jena-S3-1C** | - | - | 17.793 | 509 |
| **LB-Schmölln-1A** | 21.242 | 557 | 16.672 | 536 |
| **LB-Schmölln-1B** | 19.990 | 558 | 7.762 | 533 |
| **LB-Schmölln-1C** | 17.612 | 555 | 27.439 | 535 |
| **LB-Schmölln-2A** | 17.584 | 523 | 15.029 | 531 |
| **LB-Schmölln-2B** | 10.498 | 495 | 8.353 | 537 |
| **LB-Schmölln-2C** | 102.404 | 516 | 16.529 | 528 |
| **LB-Saalfeld-1A** | 26.082 | 561 | 23.180 | 527 |
| **LB-Saalfeld-1B** | 25.638 | 531 | 14.301 | 524 |
| **LB-Saalfeld-1C** | 24.703 | 539 | 42.882 | 520 |
| **LB-Saalfeld-2A** | 14.798 | 511 | 13.249 | 518 |
| **LB-Saalfeld-2B** | 11.503 | 484 | 12.262 | 514 |
| **LB-Saalfeld-2C** | 12.981 | 506 | 9.960 | 517 |
| **LB-Schlossv-1A** | 39.907 | 559 | 6.854 | 527 |
| **LB-Schlossv-1B** | 26.575 | 553 | 9.935 | 520 |
| **LB-Schlossv-1C** | 33.577 | 553 | 16.253 | 526 |
| **LB-Schlossv-2A** | 34.187 | 490 | 6.924 | 520 |
| **LB-Schlossv-2B** | 31.977 | 511 | 4.385 | 519 |
| **LB-Schlossv-2C** | 20.777 | 540 | 7.217 | 518 |
| **SS-Jena-1A** | 40.233 | 565 | 36.609 | 528 |
| **SS-Jena-1B** | 36.122 | 570 | 11.314 | 533 |
| **SS-Jena-1C** | 36.576 | 565 | 46.963 | 532 |
| **SS-Jena-2A** | 34.107 | 556 | 12.209 | 532 |
| **SS-Jena-2B** | 22.293 | 562 | 15.127 | 534 |
| **SS-Jena-2C** | 24.756 | 558 | 29.519 | 535 |
| **SS-Weim-1A** | 39.883 | 573 | 15.591 | 545 |
| **SS-Weim-1B** | 36.285 | 571 | 31.944 | 532 |
| **SS-Weim-1C** | 45.212 | 567 | 29.564 | 529 |
| **SS-Weim-2A** | 24.457 | 551 | 17.480 | 529 |
| **SS-Weim-2B** | 28.348 | 560 | 8.166 | 521 |
| **SS-Weim-2C** | 16.001 | 560 | 15.722 | 526 |
| **SS-Rudol-1A** | 35.503 | 573 | 11.792 | 534 |
| **SS-Rudol-1B** | 29.760 | 562 | 14.161 | 520 |
| **SS-Rudol-1C** | 25.978 | 571 | 28.028 | 531 |
| **SS-Rudol-2A** | 46.357 | 561 | 12.047 | 526 |
| **SS-Rudol-2B** | 44.304 | 560 | 33.660 | 524 |
| **SS-Rudol-2C** | 16.501 | 553 | 16.562 | 530 |
|  |  |  |  |  |
